# Dual‐Targeted Glucose‐Dependent Insulinotropic Polypeptide‐Loaded Photothermal Nanoparticles to Prevent Obesity via Lipolysis and Browning of White Adipose Tissue

**DOI:** 10.1002/smsc.202500300

**Published:** 2025-09-08

**Authors:** Ting Xie, Lutang Zhao, Shurui Pei, Kaikai Wen, Sijia Fei, Wan Chen, Zhengyang Li, Long Zhang, Linlin Li, Lixin Guo, Hui Huang, Qi Pan

**Affiliations:** ^1^ Department of Endocrinology Beijing Hospital National Center of Gerontology Institute of Geriatric Medicine Chinese Academy of Medical Sciences Beijing 100730 P. R. China; ^2^ College of Materials Science and Opto‐Electronic Technology University of Chinese Academy of Sciences Beijing 101408 P. R. China; ^3^ Beijing Key Laboratory of Micro‐Nano Energy and Sensor Center for High‐Entropy Energy and Systems Beijing Institute of Nanoenergy and Nanosystems Chinese Academy of Sciences Beijing 101400 P. R. China; ^4^ The First Affiliated Hospital Hengyang Medical school University of South China Hengyang Hunan 421001 P. R. China; ^5^ School of Chemical Engineering and Technology State Key Laboratory of Chemical Engineering and Low‐Carbon Technology Tianjin University Tianjin 300072 China

**Keywords:** obesity, photothermal therapy, targeted therapy, white adipose tissue

## Abstract

Obesity, a manifestation of energy imbalance, has become a global health pandemic. However, current pharmacological treatments, which target the central nervous system to suppress appetite and the gastrointestinal tract to inhibit nutrient absorption, have inevitably led to adverse effects, for example, depression or muscle loss. And here is limited research and development on drugs that promote energy expenditure. This study aims to construct dual‐targeted glucose‐dependent insulinotropic polypeptide (GIP)‐loaded photothermal nanoparticles (GIP/ICG@P/R8 NPs), to target white adipose tissue (WAT) and induce energy expenditure through adipose tissue remodeling. The results reveal that GIP/ICG@P/R8 NPs effectively promoted energy expenditure by inducing browning, lipolysis, and apoptosis of WAT in vitro and in vivo, outperforming single GIP or photothermal therapy. Furthermore, treatment with GIP/ICG@P/R8 NPs effectively improves systemic metabolic profiles, including a 9.23% reduction in body weight after 14 days, enhanced insulin sensitivity, and amelioration of fatty liver. Beyond that, targeted drug delivery has demonstrated favorable safety profiles, as evidenced by the absence of significant morphological or pathological changes in major organs and muscle tissues following administration. In summary, this dual‐targeted NPs platform represents a promising strategy for combating obesity and related metabolic diseases.

## Graphical abstract

Current weight‐loss medications inevitably cause adverse effects such as depression or muscle loss. The developed GIP/ICG@P/R8 NPs specifically promoted energy expenditure by inducing browning, lipolysis and apoptosis of WAT in vitro and in vivo. The results showed a 9.23% weight reduction after 14 days, enhanced insulin sensitivity, improved fatty liver condition, excellent safety profiles with no muscle decreasing.

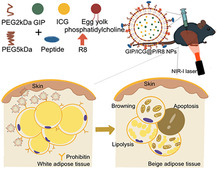

## 1 Introduction

The World Health Organization (WHO) defines overweight and obesity as a body mass index over 25 and 30 kg m^−2^, respectively. Obesity is recognized as a chronic, progressive metabolic disease with unbalanced energy absorption and consumption.^[^ ^1^ ^]^ To date, obesity has become a worldwide health problem with a substantially increasing prevalence. The WHO estimates that one in three children aged 5–9 years and one in four adolescents are affected by overweight or obesity.^[^ ^2^ ^]^ Moreover, obesity is a major risk factor for metabolic diseases, including type 2 diabetes mellitus, metabolic‐associated fatty liver disease, cardiovascular stroke, and certain cancers, and is associated with significantly increased premature morbidity and mortality.^[^ ^3^ ^]^ Currently, most Food and Drug Administration (FDA)‐approved weight‐loss medications act on the central nervous system to suppress appetite and thereby reduce energy intake. However, these treatments often exhibit limited efficacy and are associated with adverse side effects. Moreover, few studies have explored therapeutic strategies that enhance energy expenditure.^[^ ^4^, ^5^, ^6^, ^7^ ^]^ Furthermore, systemic administration carries the risk of off‐target effects and potential unforeseen adverse consequences.^[^ ^8^ ^]^ Consequently, there is an urgent need for safe and effective therapeutic approaches to promote energy expenditure to prevent obesity and its related metabolic diseases.

Adipose tissue is broadly classified into two types: white adipose tissue (WAT) and brown adipose tissue (BAT). WAT primarily functions as an energy reservoir by storing triglycerides,^[^ ^9^ ^]^ whereas BAT dissipates energy by oxidizing free fatty acids (FFAs) or glucose, thereby contributing to thermogenesis and maintaining energy balance.^[^ ^10^ ^]^ A distinct type of adipocytes, known as brown‐like or beige adipocytes, can also be found within WAT. These cells express BAT‐specific genes and proteins.^[^ ^11^ ^]^ WAT can be converted into beige adipose tissue (browning) in response to stimuli such as cold exposure, β3‐adrenergic agonists, or peroxisome proliferator‐activated receptor γ (PPARγ) agonists, thereby promoting energy consumption.^[^ ^12^, ^13^, ^14^ ^]^ Therefore, the induction of beige adipocyte biogenesis from WAT represents a promising therapeutic strategy to enhance energy expenditure for obesity management.

Glucose‐dependent insulinotropic polypeptide (GIP) is an incretin hormone that is secreted by K cells in the intestinal mucosa.^[^ ^15^ ^]^ Apart from pancreatic β‐cells, the GIP receptor (GIPR) is also expressed in the central nervous system,^[^ ^16^ ^]^ subcutaneous and visceral WAT, BAT,^[^ ^17^, ^18^ ^]^ pericytes,^[^ ^19^, ^20^ ^]^ and adipocytes,^[^ ^21^ ^]^ suggesting its potential role in regulating energy metabolism. It has been reported that, in addition to regulating postprandial glucose homeostasis, GIPRs expressed in WAT promote adipose thermogenesis and enhance energy expenditure.^[^ ^22^ ^]^ Hiradate et al. developed dual‐targeted rosiglitazone‐loaded NPs to specifically target adipocytes and minimize systemic side effects.^[^ ^23^ ^]^ However monotherapy with a single targeted agent exhibited limited therapeutic efficacy. Photothermal therapy (PTT) is a technique that converts light energy into localized heat and has been widely applied in the ablation of pathological tissues.^[^ ^24^ ^]^ Studies have shown that local photothermal stimulation at the inguinal site of mice can induce thermogenesis in beige adipocytes. Zan et al. further demonstrated that a combination of PTT of copper sulfide nanoparticles (NPs) and pharmacotherapy remodeled adipose tissue in obesity.^[^ ^25^ ^]^ However, metal photothermal agents have raised widespread concerns due to their poor clearance and long‐term biocompatibility and biosafety issues in vivo.^[^ ^26^ ^]^ Furthermore, the FDA‐approved reagent indocyanine green (ICG) as an organic photothermal reagent has recently received considerable attention for high photothermal transmission ability and less negligible toxicity.^[^ ^27^ ^]^ Based on these findings, we proposed that dual‐targeted NPs could serve as a delivery platform to selectively target WAT by coloading the organic photothermal agent ICG and GIP to combine pharmacotherapy and PTT. This strategy aims to synergistically enhance WAT browning and energy expenditure.

Therefore, dual‐targeted NPs coloaded with ICG and GIP (GIP/ICG@P/R8 NPs) were developed to investigate their antiobesity effects. The NPs were functionalized with a prohibitin‐binding peptide for WAT‐specific targeting^[^ ^28^ ^]^ and a cell‐penetrating peptide to enhance cellular uptake and intracellular transport.^[^ ^29^ ^]^ The FDA‐approved organic photothermal agent ICG enables deep tissue penetration under the first near‐infrared (NIR‐I) irradiation. The results showed that GIP/ICG@P/R8 NPs exerted superior effects in curbing obesity progression in diet‐induced obesity (DIO) mice. In addition, the NPs significantly improved the systemic metabolic profile of the treated mice. After 14 days of administration, the body weight of DIO mice was reduced by 9.23%, accompanied by improved glucose tolerance and alleviation of fatty liver. Additionally, GIP/ICG@P/R8 NPs have demonstrated favorable safety profiles, as evidenced by similar morphological or pathological manifestations between experimental and control group in major organs and muscle tissues. Furthermore, several interrelated signaling pathways were implicated in mediating these effects, including adipose tissue browning, lipolysis, and apoptosis. In summary, this study highlighted the potential of this therapy in the treatment of weight gain and associated metabolic disorders.

## 2 Results

### 2.1 Synthesis and Characterization of GIP/ICG@P/R8 NPs

**Figure 1:**
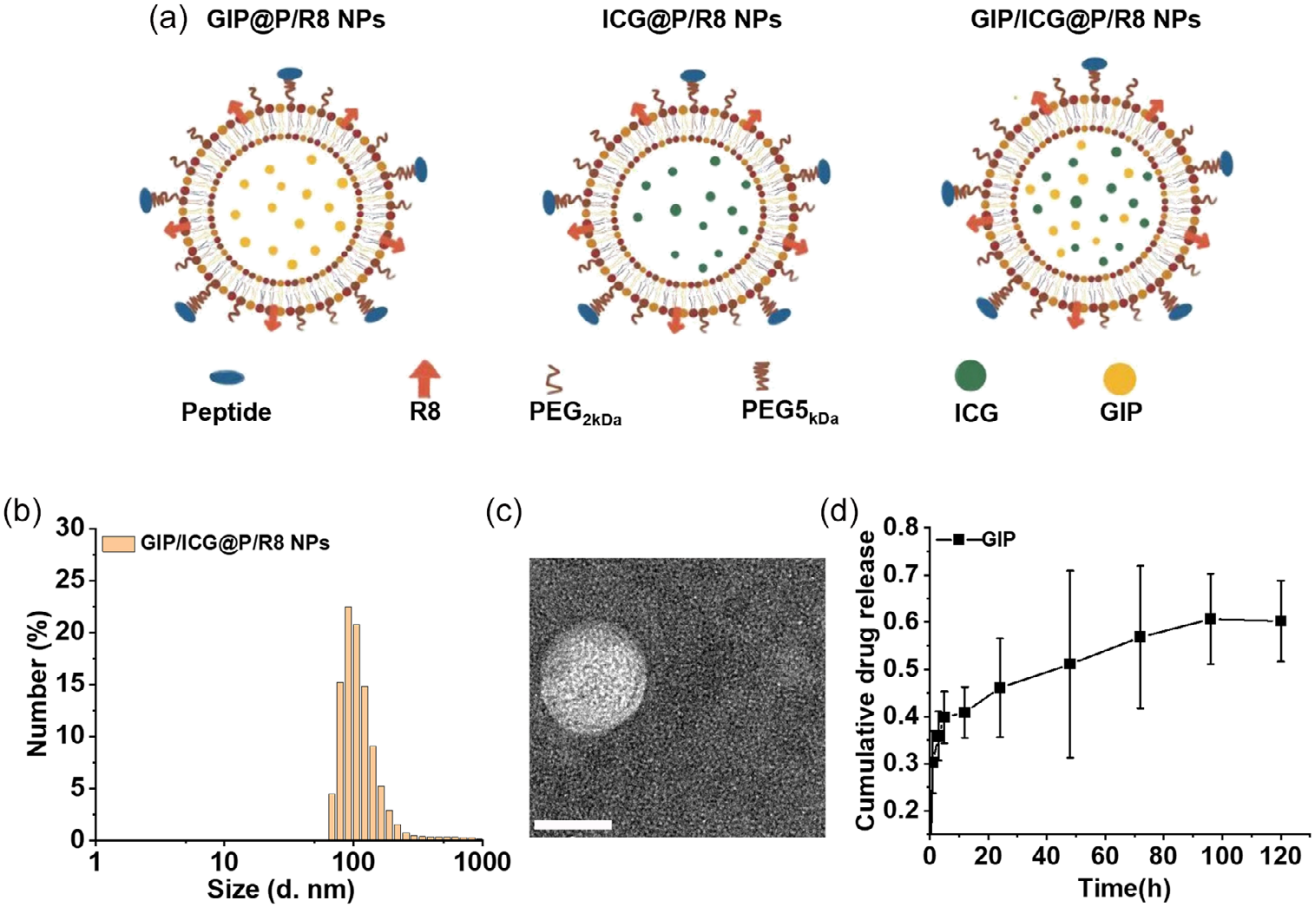
Characteristics of GIP/ICG@P/R8 NPs. a) Schematic illustration of the design of the GIP@P/R8,ICG@P/R8 and GIP/ICG@P/R8 NPs. b) The size of GIP/ICG@P/R8 NPs test by DLS. c) Transmission electron microscope of GIP/ICG@P/R8 NPs. Scale bar = 50 nm. d) Cumulative release profile of GIP from GIP@P/R8 NPs over time (n = 3).

Lipid NPs were prepared by reverse evaporation of a chloroform solution of egg yolk phosphatidylcholines and cholesterol to improve the targeting ability of these NPs, two types of PEG spacers were added, including PEG_2kDa_ and PEG5_kDa_,^[^ ^30^ ^]^ where the latter was linked to the prohibitin targeting peptide (CKGGRAKDC) which is expressed on the surface of white adipocytes or vascular endothelial cells of WAT.^[^ ^28^ ^]^ Furthermore, R8 was coated on the surface of the NPs to enhance penetration and trafficking. Subsequently, ICG and GIP were incorporated into the dual‐targeted NPs which were designated GIP/ICG@P/R8 NPs (**Figure** **1**a). GIP@P/R8 NPs and ICG@P/R8 NPs were synthesized for comparison with GIP/ICG@P/R8 NPs. Dynamic light scattering (DLS) showed that the average size, polydispersity index, and zeta potential were 123.6 nm, 0.433 and + 35.1 mV, respectively, attributed to the dual‐peptide coating (Figure 1b). Compared with naked NPs, the zeta potential of dual‐targeted NPs was changed, indicating the successful coating of P/R8 peptides (Figure S1a, Supporting Information). The morphology and size of these NPs were characterized using transmission electron microscope (Figure 1c and Figure S1b–e, Supporting Information), which showed that GIP/ICG@P/R8 NPs had a diameter of ≈64.9 nm attributable to the addition of GIP and ICG. The results further demonstrated that GIP/ICG@P/R8 NPs achieved high encapsulation efficiencies of 78.46% (ICG) and 81.35% (GIP) (Figure Sg–i, Supporting Information). Furthermore, the cumulative drug release profile revealed that the GIP release reached 45% on day 1 and peaked at 60% by day 5 (Figure 1d, Figure S1f, Supporting Information). These results demonstrated successful ICG/GIP@P/R8 NPs construction.

### 2.2 Effects of GIP/ICG@P/R8 NPs on White Adipocytes In Vitro

**Figure 2:**
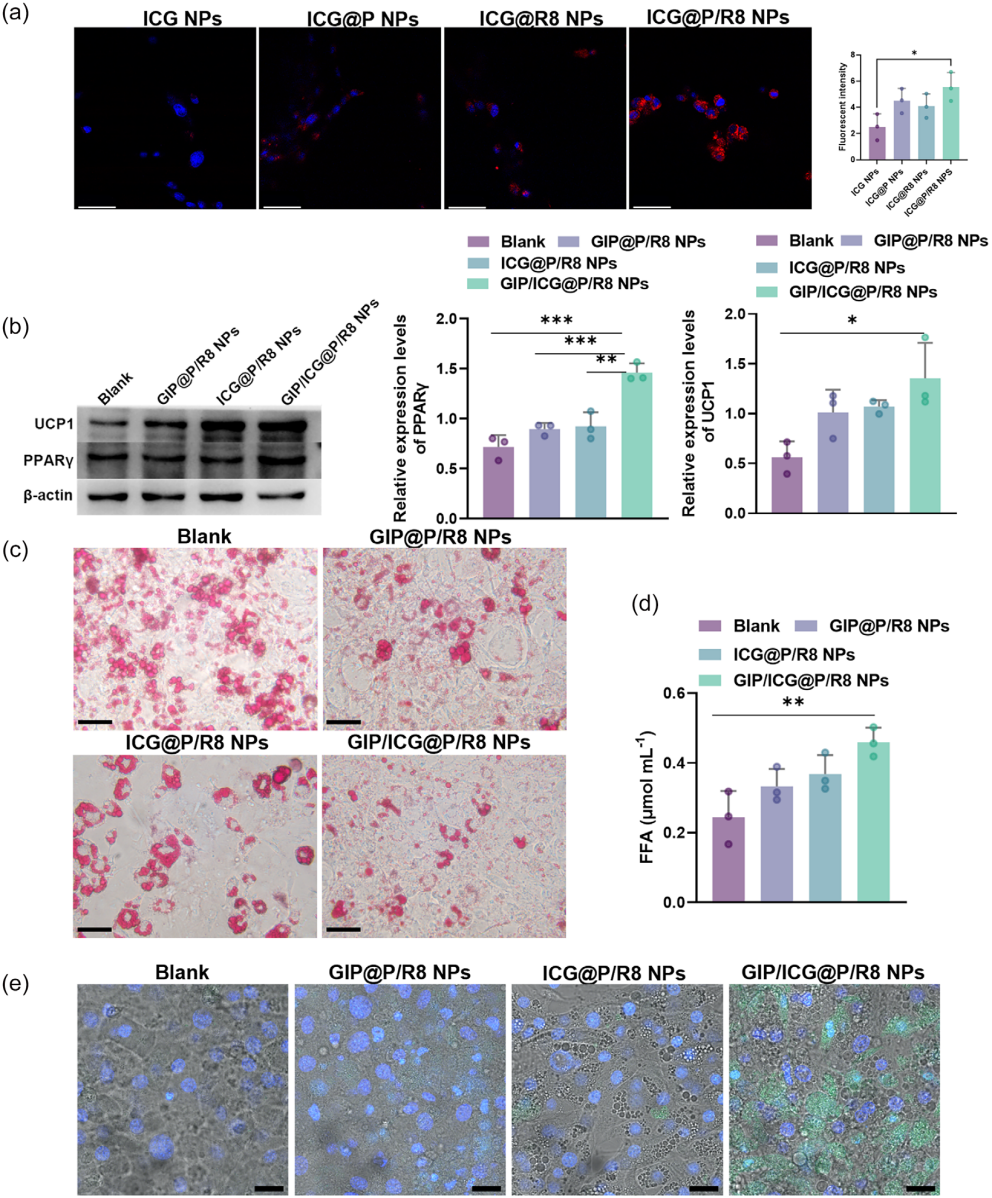
Effects of GIP/ICG@P/R8 NPs on white adipocytes in vitro. a) Confocal fluorescence images of mature white adipocytes incubated with ICG, ICG@P, ICG@R8, ICG@P/R8 NPs for 1 h. (ICG, red fluorescence). Scale bars, 58 μm (n = 3). b) Western blot analysis of UCP1 and PPARγ protein (n = 3), c) oil Red O staining of intracellular lipid droplets in white adipocytes, scale bar, 50 μm, d) quantification of FFA levels of the supernatant (n = 3), and e) confocal fluorescence images (TUNEL staining, green and DAPI, blue; scale bars, 25 μm) in mature adipocyte treatment with GIP@P/R8, ICG@P/R8 and GIP/ICG@P/R8 NPs. Data was presented as mean ± SD. Statistical significance was determined by one‐way ANOVA: *P < 0.05, **P < 0.01, ***P  < 0.001, ****P < 0.0001 (a,b,d).

Differentiated 3T3‐L1 cells were employed as an in vitro white adipocyte model^[^ ^31^ ^]^ (Figure S1j, Supporting Information). To identify the optimal drug concentration, CCK‐8 assays revealed a dose‐dependent decrease in cell viability when administrated with GIP@P/R8 NPs. Accordingly, a concentration of 0.008 mg mL^−1^ was selected for subsequent experiments of GIP, with ICG maintained at 15 μg mL^−1^ throughout the study (Figure S2a,b, Supporting Information). Then the cellular uptake of different NPs in mature 3T3L1 cells were verified by confocal laser scanning microscope. As shown in **Figure** **2**a, ICG@P/R8 NPs exerted a higher efficiency in promoting cellular uptake, which increased with time (Figure S2c, Supporting Information), demonstrating the superior specificity of dual‐targeted NPs. Subsequently, the ability of GIP/ICG@P/R8 NPs to transdifferentiate WAT into beige‐like adipocytes were tested. Quantitative real‐time PCR (RT‐qPCR) showed that after administration, the browning markers uncoupling protein 1 (UCP1) and peroxisome proliferator‐activated receptor γ (PPARγ) genes were changed (Figure S2d,e, Supporting Information), as well as the protein levels (Figure 2b), indicating the potent browning ability of dual‐targeted NPs. In order to investigate the lipolysis effects of GIP/ICG@P/R8 NPs, oil red O staining showed a significant reduction in lipid droplet size within adipocytes following treatment (Figure 2c), furthermore, the rise in FFA levels provided additional evidence for improved lipolysis efficiency (Figure 2d). Lastly, terminal deoxynucleotidyl transferase dUTP nick end labeling (TUNEL) showed that GIP/ICG@P/R8 NPs induced mild apoptosis in adipocytes (Figure 2e). In conclusion, GIP/ICG@P/R8 NPs could induce browning, lipolysis, and apoptosis in mature 3T3L1 cells in vitro.

### 2.3 Browning, Lipolysis and Apoptosis of GIP/ICG@P/R8 NPs in DIO Mice

**Figure 3:**
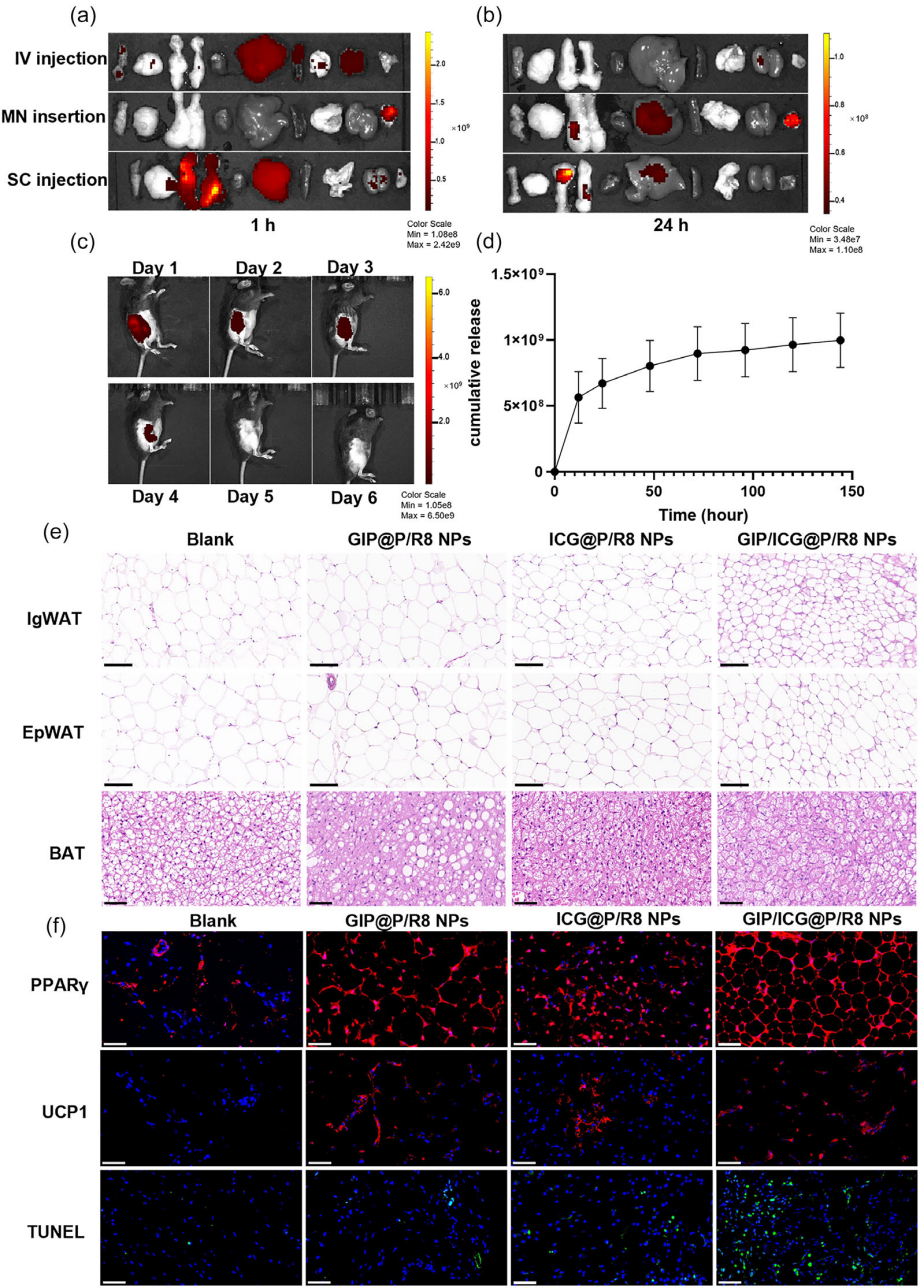
Browning, lipolysis and apoptosis of GIP/ICG@P/R8 NPs in DIO mice. a,b) Biodistribution of ICG@P/R8 NPs in major organs and adipose tissues at 1 h (a) and 24 h b) following IV injection, microneedle (MN) insertion, or local subcutaneous (SC) injection of ICG@P/R8 NPs solution (50 μL, 0.5 mg mL^−1^ of ICG). c) Longitudinal in vivo fluorescence images and d) cumulative release profile from Day 1–Day 6 after SC injection of GIP/ICG@P/R8 NPs (n = 3). e) H&E staining images of inguinal WAT (IgWAT), epididymal WAT (EpiWAT), and BAT. Scale bars = 0.1 mm (IgWAT and EpiWAT), 0.05 mm (BAT). f) PPARγ‐labeled (red) and UCP1‐labeled (red) immunofluorescence images of IgWAT and TUNEL staining (green) of IgWAT after different treatment. Nuclei were counterstained with DAPI (blue). Scale bar = 0.05 mm.

Next, a suitable route of administration was explored. Compared to intravenous injection (IV), local subcutaneous (SC) administration with an insulin microneedle in the inguinal region of mice showed that almost 47.8% of the GIP/ICG@P/R8 NPs remained in the inguinal region 1 h after administration, while only 3.6% remained in the inguinal region following IV injection. After 24 h, 52.6% remained in the inguinal WAT (IgWAT) via the SC route while there was little fluorescence in the IgWAT via the IV route (**Figure** **3**a,b). A polymeric microneedle (MN) patch was reported to deliver medications with the advantages of minimal invasiveness and high bioavailability.^[^ ^32^ ^]^ However, only 19.4% reached the IgWAT by diffusion after 24 h and 42.6% remained on the surface of the skin, which could cause a burning sensation during PTT. Therefore, local SC injection into IgWAT was chosen as the administration method in subsequent studies. Furthermore, in vivo imaging system revealed that the release curve of GIP/ICG@P/R8 NPs was ≈4 days (Figure 3c, d). The whole‐body distribution and fat uptake of GIP/ICG@P/R8 NPs were evaluated by Living Image (Figure S2f, Supporting Information). We observed that after administration of 5 min, the high initial uptake of GIP/ICG@P/R8 NPs in IgWAT, EpiWAT, and liver of mice. After 1 h, the fluorescence intensity of IgWAT and liver attained maximal levels and then gradually reduced.

**Figure 4:**
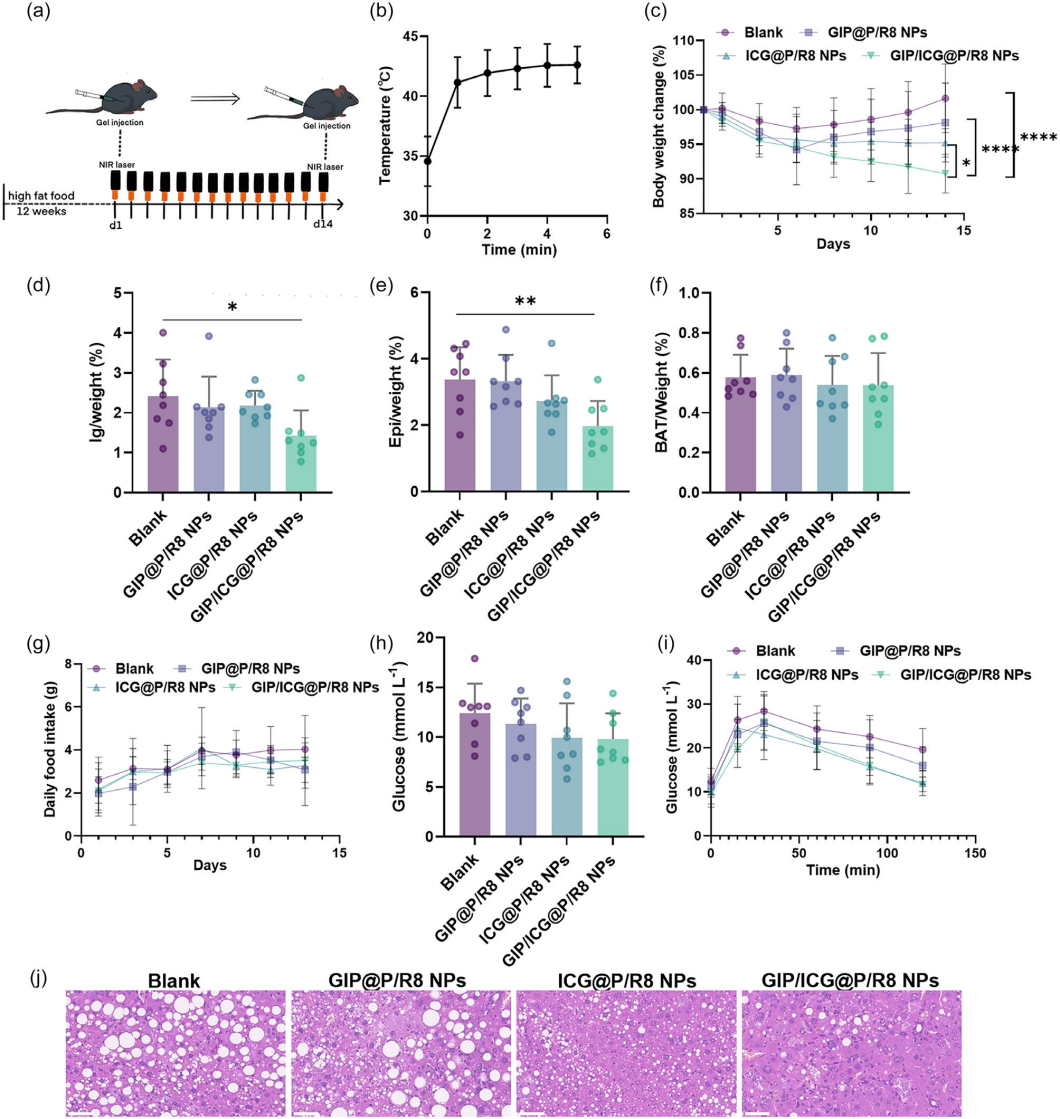
Systemic effects of GIP/ICG@P/R8 NPs. a) Schematic illustration of the treatment protocol involving a 12‐week HFD followed by NPs administration and PTT. b) Temperature elevation curve of SC injection of ICG‐containing NPs followed by the NIR‐I laser irradiation (n = 8). c) Changes in body weight over 14 days of treatment (n = 8). d–f) Weight ratios of IgWAT, EpiWAT, and BAT relative to total body weight (n = 8). g) Daily food intake during the treatment period (n = 8). h,i) Serum glucose and glucose sensitivity change after different treatments (n = 8). j) H&E staining images of liver cells. Scale bar = 0.05 mm. Data represent the mean ± SD. Statistical analysis was performed using one‐way ANOVA for (d–f,h) and two‐way ANOVA for (c,g,i): *P < 0.05, **P < 0.01, ***P < 0.001, ****P < 0.0001.

Subsequently, DIO mice were used to evaluate the effects of GIP/ICG@P/R8 NPs on WAT in vivo. After 14 days of treatment (**Figure** **4**a), SC WAT (IgWAT), and visceral WAT (epididymal WAT, EpiWAT), and BAT from the scapula area were dissected. Hematoxylin‐eosin (HE) staining images showed that the IgWAT, EpiWAT of SC injection of GIP/ICG@P/R8 NPs exhibited smaller lipid droplets than that of ICG@P/R8 or GIP@P/R8 NPs (Figure 3e), indicating the strongest lipolysis ability of GIP/ICG@P/R8 NPs. Subsequently, immunofluorescence images showed that the upregulation of UCP1 and PPARγ of IgWAT in mice SC injection of GIP/ICG@P/R8 NPs was more obvious than single therapy, suggesting the superior browning ability of synergistic GIP/ICG@P/R8 NPs (Figure 3f). Furthermore, GIP/ICG@P/R8 NPs resulted in increased apoptosis, which was consistent with the results of adipocytes in vitro. Collectively, these findings indicated that GIP/ICG@P/R8 NPs were more efficient both in vitro and in vivo.

### 2.4 Systemic Effects of GIP/ICG@P/R8 NPs

Considering the browning and lipolysis ability of GIP/ICG@P/R8 NPs in adipocytes, it is worthwhile testing their metabolic performance on the whole body in vivo. Therefore, DIO mice which had been given a high‐fat diet (HFD) for 12 weeks were constructed. The mice were then divided into four groups with similar initial weight (Figure S2g, Supporting Information), and were SC injected with PBS, GIP@P/R8, ICG@P/R8, and GIP/ICG@P/R8 NPs in bilateral IgWAT, followed by the NIR‐I laser irradiation of 5 min for the last two groups and repeated for 14 days, and were continued on a HFD for 14 days (Figure 4a).

For the mice of GIP/ICG@P/R8 NPs and ICG@P/R8 NPs groups, the skin temperature in the inguinal region of DIO mice was between 42 and 45 °C (Figure 4b, Figure S2h, Supporting Information). Notably, body weight of DIO mice declined by 9.23% after administration (Figure 4c), an effect that persisted regardless of NIR or ICG alone (Figure S2i, Supporting Information). Moreover, not only the IgWAT shrink significantly, but the EpiWAT also showed significant decreases, and BAT showed no significant changes (Figure 4d–f). Moreover, food intake was similar among the four groups (Figure 4g), eliminating difference in energy absorption. Apart from antiobesity effects, the GIP/ICG@P/R8 NPs group also showed better metabolic fitness with decreased fasting glucose and improved glucose sensitivity (Figure 4h,i), and also a decreasing tendency of serum lipid levels (Figure S3a–c, Supporting Information). Interestingly, H&E staining images revealed that lipid droplets in liver cells became smaller and fewer, and fatty liver improved accordingly (Figure 4j). In addition, normal chow‐fed DIO mice were also investigated and it was found that after 14 days, the mice SC injected with GIP/ICG@P/R8 NPs and followed by the NIR‐I laser irradiation also inhibited obesity development with a 23.48% weight reduction, in line with the previous examination. Additionally, this treatment resulted in the lowest glucose, total cholesterol, and low‐density lipoprotein levels (Figure S3d–i, Supporting Information). Taken together, these findings suggest dual‐targeted GIP‐loaded photothermal NPs have the potential to treat obesity and related metabolic dysfunctions.

### 2.5 Biocompatibility of GIP/ICG@P/R8 NPs

**Figure 5:**
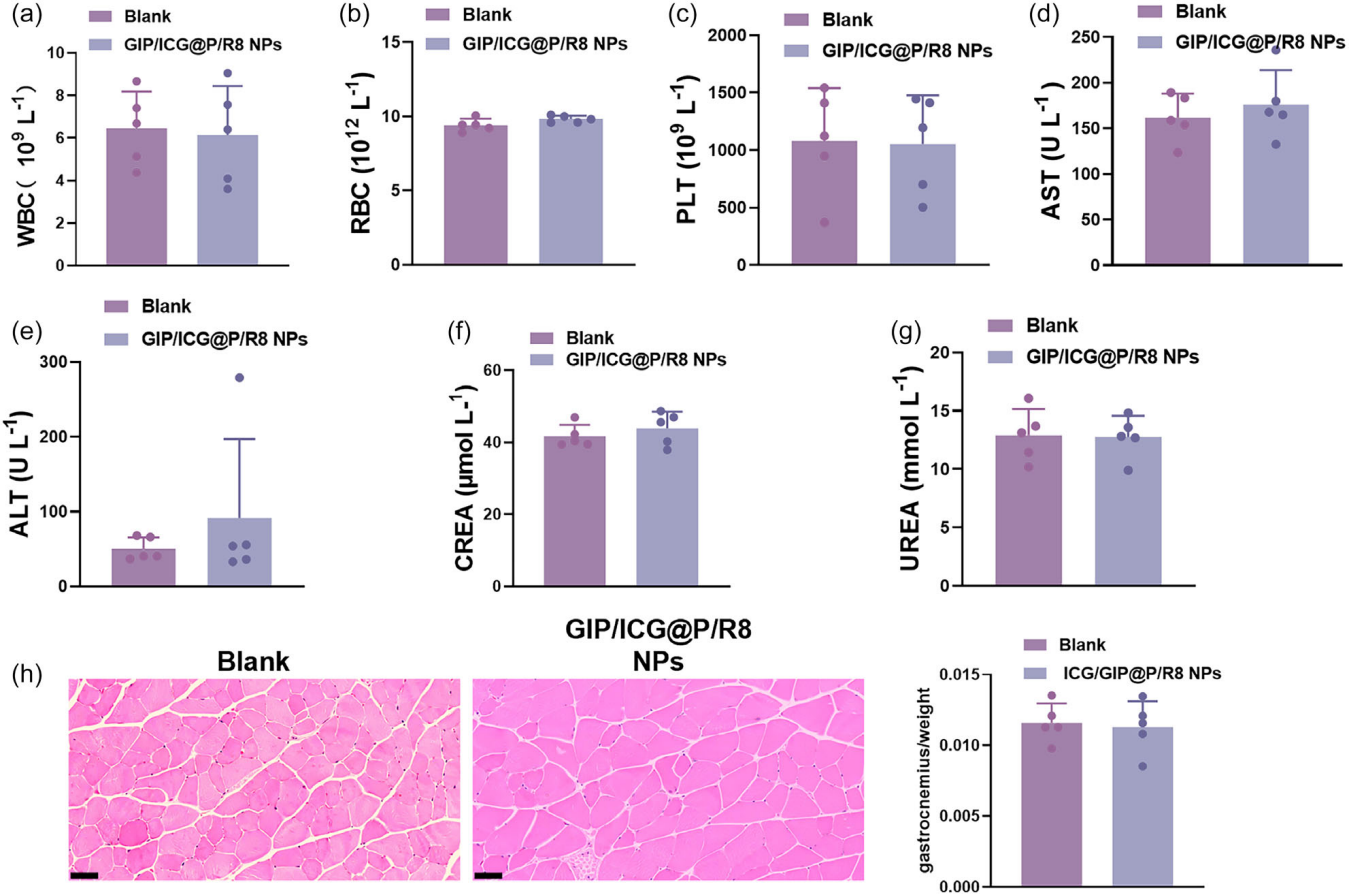
Biocompatibility assessment of GIP/ICG@P/R8 NPs in vivo. a–c) Serum parameters of white blood cells (WBC), red blood cells (RBC), and platelets (PLT) (n = 5). d–g) Hepatic and renal function markers, including alanine aminotransferase (ALT, U L^−1^), aspartate aminotransferase (AST, U L^−1^), creatinine (CREA, μmol·L^−1^), and urea (mmol·L^−1^) (n = 5). h) H&E staining of the gastrocnemius and quantitative assessment of muscle‐to‐body weight ratio after different treatments (n = 5). Scale bar = 0.05 mm. Data represent the mean ± SD (n = 5). Statistical significance was determined by unpaired two‐tailed Student's *t*‐test.

To systematically assess the biosafety profile of GIP/ICG@P/R8 NPs, comprehensive safety evaluations were conducted in murine models, encompassing serum biochemical analysis and histopathological examination. The results showed that after 14 days treatment, serum parameters showed no obvious changes (**Figure** **5**a–c). Additionally, hepatic and renal parameters (Figure 5d–g) remained comparable to those in the control group. Subsequent organ histology revealed that there was no detectable hepatic, renal, lienal, pulmonary, or cardiac necrosis, congestion, or hemorrhage, and no morphological changes (Figure S4a,b, Supporting Information). Furthermore, previous drugs, which mainly reduce fat through central appetite suppression, inevitably reduce the corresponding muscle tissue.^[^ ^33^ ^]^ Accordingly, histological analysis was extended to the gastrocnemius muscle, and there were no significant differences in muscle weight and morphology after administration (Figure 5h). Taken together, these results demonstrated the superior biocompatibility of GIP/ICG@P/R8 NPs, providing a new and promising option for safe and effective treatment of weight loss.

## 3 Discussion

In this study, we developed dual‐targeted photothermal‐pharmacotherapy that achieved a significant 9.23% reduction in body weight in DIO mice while improving glucose tolerance, without inducing observable muscle decreasing. Notably, hepatic lipid accumulation was significantly alleviated, as evidenced by smaller and fewer lipid droplets in hepatocytes attributed to adipocyte thermogenesis, lipolysis, and apoptosis.

The GIP/ICG@P/R8 NPs exhibited high encapsulation efficiencies of 78.46% (ICG) and 81.35% (GIP). ≈55% of GIP was released by day 4 in a sustained manner. The NPs were functionalized with dual‐targeted, high‐affinity peptides, including a prohibitin‐binding peptide and octaarginine (R8). The prohibitin‐binding peptide has been reported to be specifically expressed on the surface of white adipocytes and vascular endothelial cells in WAT.^[^ ^34^ ^]^ R8, a well‐known cell‐penetrating peptide, facilitates enhanced cellular uptake and intracellular transport.^[^ ^29^ ^]^ Laser scanning confocal microscope showed that, compared with nonmodified NPs, dual‐targeted NPs showed significantly increased cellular internalization, further confirming the successful construction and efficient targeting capability of this delivery system.

To elucidate the in vitro and in vivo bioactivity of GIP/ICG@P/R8 NPs, we systematically evaluated their potential to induce white adipocyte remodeling via browning, lipolysis, and apoptosis. Differentiated 3T3‐L1 murine adipocytes were selected to examine white adipocytes in vitro, and nondiabetic, genetically wild‐type, DIO mice were used as the in vivo model. The results demonstrated that both the PTT and GIP alone could induce browning, lipolysis, and apoptosis both in vitro and in vivo. Furthermore. GIP/ICG@P/R8 NPs had higher efficiency. The browning marker of UCP1, which is expressed in the mitochondria for heat production, was up‐regulated both at the mRNA and protein level after administration. PPARγ, a member of the nuclear receptor superfamily, forms a heterodimer with the retinoid X receptor (RXR) upon activation by its specific ligand. The heterodimer of PPAR–RXR translocates to the nucleus and binds to peroxisome proliferator reaction elements on DNA to regulate the transcription of target genes.^[^ ^35^ ^]^ However, the results showed that the expression pattern of PPARγ, which has recently attracted considerable attention due to its role in energy homeostasis, differed between the mRNA and protein levels. This discrepancy may be related to transcriptional regulation of PPARγ, where gene expression changes occur more rapidly than corresponding changes at the protein level.^[^ ^36^ ^]^

To further investigate the effects of GIP/ICG@P/R8 NPs on weight loss and systemic metabolism, the three types of NPs were subcutaneously injected into the IgWAT of DIO mice. The mice treated with GIP/ICG@P/R8 NPs exhibited a significantly greater reduction in body weight. Furthermore, quantitative analysis revealed that GIP/ICG@P/R8 NPs administration led to significant improvements in both glucose homeostasis and lipid profiles in the treated DIO mice. Additionally, fatty liver was alleviated, as evidenced by smaller and fewer lipid droplets in the hepatocytes. R8‐modified liposomes tend to accumulate in the liver to be recognized by opsonin or macrophages.^[^ ^37^ ^]^ Furthermore, GIP/ICG@P/R8 NPs showed excellent safety profiles, as evidenced by similar visceral organ morphology and mass.

Previous studies have shown that activation of the GIPR in adipose tissue enhances local lipid oxidation and energy expenditure, thereby promoting a preferential shift toward lipid utilization as the primary energy source.^[^ ^22^ ^]^ These findings independently confirmed the role of GIP in promoting energy expenditure. However, the overall effect of dual‐targeted GIP‐loaded NPs in promoting energy dissipation and improving systemic metabolism appeared to be limited. Several possible reasons may account for this observation. First, GIP may be rapidly inactivated by Dipeptidyl Peptidase IV (DPP‐IV), a type of enzyme which breaks down GIP, despite the sustained‐release properties of the NPs formulation.^[^ ^38^ ^]^ Second, this may be related to the targeting characteristics of the NPs, which may limit their ability to exert appetite‐suppressing effects via GIPR in the central nervous system, thereby reducing the overall therapeutic efficacy of GIP to some extent.^[^ ^39^ ^]^ This constitutes a pivotal question awaiting exploration in our follow‐up studies.

Currently, there are numerous nanoparticle‐based targeted delivery therapies for weight reduction, primarily focused on promoting thermogenesis in adipose tissue and mitigating inflammation.^[^ ^7^, ^40^, ^41^ ^]^ Regarding the enhancement of energy expenditure in adipose tissue, both ICG and GIP can synergistically stimulate cellular thermogenesis through classical and nonclassical pathways. It is currently widely accepted that there are two primary thermogenic pathways: the classical UCP1‐dependent thermogenic pathway and the UCP1‐independent thermogenic pathway.^[^ ^42^, ^43^, ^44^ ^]^ The ICG photothermal pathway belongs to UCP1‐dependent thermogenesis.^[^ ^45^, ^46^ ^]^ Mild photothermal stimulation has been shown to exert multiple metabolic benefits through several interrelated mechanisms. For instance, the transient receptor potential vanilloid 1 (TRPV1) ion channel can be activated when the local temperature exceeds 42 °C, subsequently inducing the expression of PPARγ, as a nuclear receptor transcription factor directly binds to the promoter or enhancer regions of the UCP1 gene to promote its transcription which promotes heat production.^[^ ^25^ ^]^ Beyond that, induced PPARγ by ICG may also activate the GIPR.^[^ ^47^ ^]^ In addition, PTT can modulate the local adipose microenvironment by dilating vessels, thereby increasing blood flow and alleviating hypoxia.^[^ ^48^ ^]^ In our experimental design, biocompatibility, biodegradability, and high photothermal conversion efficiency were established as critical selection criteria for the optimal photothermal agent. Zan et al. employed copper sulfide NPs, which demonstrated high photothermal conversion efficiency. However, concerns have been raised regarding the poor clearance and long‐term toxicity of inorganic nanomaterials.^[^ ^25^ ^]^ Therefore, ICG, an FDA‐approved organic NIR agent, was selected as the photothermal reagent in our NPs system. For GIP, our findings demonstrated its intervention could promote WAT thermogenesis via the UCP‐1 pathway. In contrast, studies have demonstrated that GIP can promote lipid oxidation, thermogenesis, and energy expenditure in WAT through a noncanonical thermogenic pathway‐calcium futile cycling.^[^ ^22^ ^]^ It is established that calcium futile cycling refers to a process where cells continuously consume ATP through repeated transmembrane calcium ion transport without performing effective mechanical work or signal transduction functions.^[^ ^49^ ^]^ The primary mechanism of GIP‐induced calcium futile cycling involves increased sarcolipin expression and downregulation of SERCA1 in white adipocytes. Sarcolipin competitively binds to SERCA1 with cytoplasmic calcium ions, thereby inhibiting calcium transport into the endoplasmic reticulum.^[^ ^50^, ^51^ ^]^ This process forces SERCA1 to hydrolyze more ATP.^[^ ^52^ ^]^ In summary, our engineered GIP/ICG@P/R8 NPs can simultaneously maximize adipose tissue energy expenditure through both classical and nonclassical thermogenic pathways.

## 4 Conclusions

The developed dual‐targeted photothermal‐pharmacotherapy NPs, which combined targeted peptides, an organic photothermal agent, and GIP, not only targeted WAT precisely but also synergistically promoted energy dissipation by inducing browning, lipolysis, and apoptosis of adipocytes. This strategy effectively reduced obesity and improved glucose and glucose tolerance, accompanied by favorable changes in serum metabolic parameters, which were less pronounced with single PTT or GIP alone NPs. In addition, the NPs formulation illustrated excellent safety, as confirmed by serological and histopathological assessments of related organs and muscles. Overall, these findings suggest a promising and safe therapeutic approach for combating obesity and related metabolic disorders.

## 5 Experimental Section

The experimental section is available in the Supporting Information. All animal studies were approved by the Institutional Animal Care and Use Committee at Institute of Zoology, Chinese Academy of Sciences (IOZ‐IACUC‐2024‐183).

Statistical analysis: All experiments were performed at least three times, quantitative results are presented as the mean ± standard deviation (mean ± SD), indicated by error bars in all graphs. Statistical analyzes were conducted using GraphPad Prism 9.5 and Origin 2024. Unpaired two‐tailed Student's *t*‐test were used when two groups were compared. One‐way analysis of variance (ANOVA) was used to compare the mean values of three or more groups with one independent variable. Two‐way ANOVA was used to compare the mean values three or more groups with two independent variables. A P value of less than 0.05 was considered statistically significant (*P < 0.05, **P < 0.01, ***P < 0.001, ****P < 0.0001).

## Supporting Information

Supporting Information is available from the Wiley Online Library or from the author.

## Conflict of Interest

The authors declare no conflict of interest.

## Author Contributions

**Ting Xie**: formal analysis (lead); investigation (lead); visualization (lead); writing—original draft (lead). **Shurui Pei**: data curation (equal); investigation (lead). **Lutang Zhao**: data curation (equal); formal analysis (equal); investigation (lead). **Kaikai Wen**: conceptualization (equal); data curation (equal); formal analysis (equal); investigation (lead); writing—review and editing (equal). **Sijia Fei**: conceptualization (equal); data curation (equal); methodology (equal); writing—review and editing (equal). **Wan Chen**: data curation (supporting); formal analysis (supporting). **Zhengyang Li**: data curation (supporting); formal analysis (supporting). **Long Zhang**: formal analysis (supporting); investigation (supporting). **Linlin Li**: formal analysis (supporting). **Lixin Guo**: conceptualization (lead). **Hui Huang**: conceptualization (lead); funding acquisition (equal). **Qi Pan**: conceptualization (lead); data curation (lead); formal analysis (lead); funding acquisition (lead); writing—review and editing (lead). **Ting Xie**, **Shurui Pei**, **Lutang Zhao**, and **Kaikai Wen** contributed equally to this work.

## Supporting information

Supplementary Material

## Data Availability

The data that support the findings of this study are available from the corresponding author upon reasonable request.
