# Supplementary material for: Dual‐Targeted Glucose‐Dependent Insulinotropic Polypeptide‐Loaded Photothermal Nanoparticles to Prevent Obesity via Lipolysis and Browning of White Adipose Tissue
Source: Small Sci. 2025 Sep 8;5(11):2500300. doi: 10.1002/smsc.202500300 (PMC12622501; doi:10.1002/smsc.202500300)
Supplement: Supplementary file 1 — Supplementary Material [file SMSC-5-2500300-s001.pdf]

Supporting Information

Dual-Targeted Glucose-Dependent Insulinotropic Polypeptide-Loaded Photothermal Nanoparticles to Prevent Obesity via Lipolysis and Browning of White Adipose Tissue

Ting Xie[1#], Lutang Zhao[2#], Shurui Pei[2#], Kaikai Wen[3#*], Sijia Fei[1], Wan Chen[4], Zhengyang Li[4], Long Zhang[2], Linlin Li[3], Lixin Guo[1*], Hui Huang[2,5*], Qi Pan[1*]

[#]These authors contributed equally to this work.

[1]Department of Endocrinology, Beijing Hospital, National Center of Gerontology; Institute of Geriatric Medicine, Chinese Academy of Medical Sciences, Beijing 100730, P.R. China

[2]College of Materials Science and Opto-Electronic Technology, University of Chinese Academy of Sciences, Beijing 101408, P. R. China

[3]Beijing Key Laboratory of Micro-Nano Energy and Sensor, Center for High-Entropy Energy and Systems, Beijing Institute of Nanoenergy and Nanosystems, Chinese Academy of Sciences, Beijing, 101400, P. R. China

[4]The First Affiliated Hospital, Hengyang Medical school, University of South China, Hengyang, Hunan, 421001, P.R. China

[5]School of Chemical Engineering and Technology, State Key Laboratory of Chemical Engineering and Low-Carbon Technology, Tianjin University, Tianjin 300072, China.

1.Materials and methods

1.1 Reagents

Egg yolk phosphatidylcholine (EPC) was purchased from Ponsure Biotechnology. 1,2-Distearoyl-sn-glycero-3-phosphoethanolamice N-[(3-maleimide-1-oxopropyl) aminopropyl polyethylene glycolcarbamyl] (DSPE-PEG2kDa-Mal and DSPE-PEG5kDa-Mal) were purchased from the Shanghai Aladdin Biochemical Technology. Cholesterol, insulin from bovine pancreas, dexamethasone (DEX), 3-isobutyl-1-methylxanthine (IBMX), and (−)-isoproterenol hydrochloride were purchased from Sigma Aldrich. Stearyl-octa arginine (STR-R8, abbreviated as R8) was purchased from GenScript Biotech Corporation. The prohibitin-targeting peptide (NH_2-GKGGRAKDGGC-Amide) and the adipocyte-homing peptide (CNFGHVGGC) were purchased from GenScript Biotech Corporation. Indocyanine Green (ICG， l0535-1g) was purchased from Wokai Biotechnology Co., Ltd and glucose-dependent insulinotropic polypeptide (GIP, 25004) was obtained from Cayman.

1.2 Synthesis of ICG/GIP/@P/R8 NPs

Lecithin (24 mg, 0.03 mmol) and cholesterol (3.096 mg, 0.008 mmol) were added into chloroform solution (2 mL) to obtain lipid solution, then diisopropyl ether (2 mL) and DSPE-PEG2kDa (0.4 mg, 0.146 μmol) were added to form total lipid solution. Adipocyte homing peptide solution (10 μmol) was mixed with DSPE-PEG5kDa-Mal polymer solution (10 μmol) at a ratio of 1:1, and the first mixture (1 mL) was obtained after oscillating at 30 °C for 24 h. R8 solution (4 mg) was added into HEPES solution (10 mM, 5.33 mL). The lipid solution was mixed with/without R8 (0.489 mmol L^{-1}, 2 mL), the first mixture (0.05 mL, 0.5 μmol)，or the same amount of R8 (0.489 mmol L^{-1}, 2 mL) and the first mixture (0.05 mL, 0.5 μmol), and then well mixed to obtain the second mixture. In this process, non-targeted, single-targeted, and dual-targeted NPs could be constructed for subsequent experiments. Then, ICG (1 mg), GIP (5 mg) or the same amount of ICG (1 mg) and GIP (5 mg) was added to the second mixture to obtain ICG@P/R8 NPs, GIP@P/R8

NPs, GIP/ICG@P/R8 NPs, respectively, and then it was homogenized by ultrasonic cell breaker for 10 min after stirring and treated by ultrasonic detergent (SCIENTZ, 75W) for 3 min after spinning the organic solvent to obtain NPs suspension. Final products were obtained after dialysis of the NPs suspension with HEPES buffer (pH 7.4,10 mM) for 2 h.

1.3 Characterization of GIP@P/R8, ICG@P/R8, and GIP/ICG@P/R8 NPs

The size and zeta potential of GIP@P/R8, ICG@P/R8, and GIP/ICG@P/R8 NPs were tested by a dynamic light scattering instrument (DLS, Zetasizer ZS90). The content of ICG and GIP was quantified by a UV-visible spectrophotometer (Agilent Cary 60). The cargo loading rate was tested by comparing the content before and after dialysis. The release curve was calculated by measuring the absorption curve of the drug outside the dialysis bag released from each NPs in the dialysis bag at a preset time.

1.4 Cell cultures

3T3L1 preadipocytes were cultured with 3000 cells cm^{-2} in basal medium containing Dulbecco's Modified Eagle's Medium (DMEM) supplemented with 10% fetal bovine serum (FBS), 100 μmol mL^{-1} penicillin streptomycin, and 1 mmol sodium pyruvate at 37 °C in 5% CO_2 incubator. They were incubated for three days (from day 0 to day 2) in a differentiation medium based on basal medium (10 μg mL^{-1} insulin, 0.25 μg mL^{-1} DEX, and 0.5 mM IBMX) to differentiate into mature adipocytes. From day 3 to day 10, the cells were further maintained in a insulin medium based on basal medium containing 10 μg mL^{-1} insulin. The insulin medium was changed at two-day intervals. Mature adipocytes were distinguished from preadipocytes by assessing the morphology. On day 10, 12, and 14, the cells was treated with ICG@P/R8 NPs (ICG, 15 μg mL^{-1}) with NIR for 5 minutes, GIP@P/R8 NPs (GIP, 30 μmol L^{-1}) and GIP/ICG@P/R8 NPs (15 μg mL^{-1} of ICG, 30 μmol L^{-1} of GIP) with NIR for 5 minutes, respectively. On day 15, the cells were treated with isoproterenol (10 μmol, 10 mM HEPES buffer) for 5 hours.

1.5 Cell viability

The 3T3L1 pellet was resuspended in 100 μL extracellular matrix (ECM) without PBS, incubated in 96-well plates for 1 d, and treated with different concentrations of GIP@P/R8 NPs and ICG@P/R8 NPs. Cell viability was assessed by the Cell Counting Kit-8 (CCK-8) assay (14450-1-AP, Proteintech), and were incubated for 0.5 h (37 °C, 5% CO_2). Then the absorbance at 450 nm was measured (Thermo Fisher).

1.6 Quantification of *in vitro* cellular uptake of NPs

Confocal laser scanning microscopy (Leica Microsystems CMS GmbH) was used to assess the uptake of different NPs. 3T3L1 preadipocytes ($1*10^4$ cells well^{-1}) were seeded in confocal dish lower. After differentiation into mature adipocytes, the dipocytes were treated with ICG, ICG@P, ICG@R8, and ICG@P/R8 NPs (ICG, 15 μg mL^{-1}) at 37°C for 1 h and 3 h. After that, the cells were washed three times with 20 μmol mL^{-1} heparin in phosphate buffer saline (PBS), and subsequently analyzed using confocal laser scanning microscopy (excitation light at 638 nm, and emission light at 805 nm).

1.7 Apoptosis of adipocytes

On day 15, apoptotic cells were detected using a TUNEL kit (Yeasen, 40306ES50). The adherent cells were washed three times with PBS and fixed with 4% paraformaldehyde for 30 minutes, followed by another three washes with PBS. PBS containing 0.3% Triton X-100 was added and incubated at room temperature for 5 minutes, followed by three washes with PBS. TUNEL detection solution was prepared: TdT enzyme (2 μL) and fluorescent labeling solution (48 μL). The samples were washed twice with PBS, and cocultured with the TUNEL detection solution for 60 minutes at 37 °C in the dark. After washing with PBS three times, the cells were encapsulated with anti-fluorescence quencher and observed by confocal laser scanning microscopy (Zeiss, LSM880).

1.8 Lipolysis ability

On day 15, the adipocytes were first treated with oil red O fixing solution (100 μL well^{-1}) for 30 min at room temperature, 60% isopropyl alcohol (150 μL) was added to each well and incubated for 5 min. Oil red O dyeing solution A and oil Red O dyeing solution B were mixed in a ratio of 3:2 to obtain the oil red O dyeing solution. After washing by PBS, the adipocytes were incubated with oil red O dyeing solution (60 μL well^{-1}) for 30 min at room temperature and washed again. The red stained lipid droplets can be visualized under an inverted fluorescence microscope (Olympus，CKX53)and photographed.

To evaluate the fatty acid levels, during the administrations, on day 12, 14 and 15, the supernatant was extracted from the 6-well plate, and determined by the FFA kit (Beyotime Biotechnology, AC 10173).

1.9 Quantitative Real-time polymerase chain reaction (Rt-qPCR) assay

On day 15, total RNA was extracted from the adipocytes by a RNAeasy™ Animal RNA Isolation Kit with Spin Column (Beyotime, R0026), and the concentration of nucleic acid was determined by spectrophotometer (Thermo, Nanodrop One C). After that, reverse transcriptase (TakaRa RR036A) and sterile enzyme-free water were added into the 10 μL system to reverse transcribe the RNA into cDNA. Furthermore, after cDNA was obtained, the last step was preparing a real-time quantitative PCR reaction of 20 μL system. The SYBR dye method was used for real-time quantification (Accurate biology, AG11701), the primer was purchased from Sangon Biotech., and the point plate operation was carried out on the 96-well plate. After running a standard-length TaqMan qPCR (QuantStudio 6 Flex), the corresponding CT (cycle threshold) were obtained (ΔΔCt).

1.10 Western blot

On day 15, the protein was extracted from the adipocytes. After denaturing the protein samples, run at 80 V in the stacking gel and then turned to 120 V in the resolving gel. Then transferred the protein to the PVDF membrane. After that, the

membrane was blocked by 5% milk at room temperature for 1 h and then added with primary antibody at 4 °C overnight (Proteintech, 16643-1-AP and 23673-1-AP, Beyotime, AF5003). Furthermore, TBST was used for cleaning PVDF membrane, and the secondary antibody was used at room temperature for 1 h (Abcam, ab97051), and TBST was used to wash the membrane for three times. Then the membrane was imaged by gel imaging system (Bio-Rad).

1.11 Animal model construction

C57BL/6 male mice purchased from Charles River aged 7-8 weeks (average weight 25 g) were reared in an SPF environment with 12-hour cycle lighting, temperature controlled at 23.5 °C, and humidity of 34 ± 5 %. The obese model was constructed with a high-fat diet (XTHF60, 5.22 kCal g^{-1}) throughout the experiments. After 3 months of feeding, the weight of the obese mouse was over 35 g on average.. During the first treatment, all the DIO mice were continued to high fat diet (HFD), while in the second time of treatment, the previously DIO mice were fed standard chow during the experiment. All animal studies were approved by the Institutional Animal Care and Use Committee in Institute of Zoology, Chinese Academy of Sciences (IOZ-IACUC-2024-183).

1.12 Biodistribution *in vivo*

To reveal the biodistribution of NPs delivered by different methods, the normal diet mice were subcutaneously injected in the inguinal region, intravenously injected, and injected with a microneedle (MN) patch with ICG@P/R8 NPs of 50 μL (0.5 mg mL^{-1}). After 1 h or 24 h of administration, fat tissues and major organs (inguinal WAT, epididymis WAT, interscapular BAT, kidney, heart, lung, liver, spleen, and skin on application sites) were visualized by an in vivo imaging system (the excitation light at 780 nm, emission light at 805 nm). To investigate the metabolism profiles, mice were subcutaneously injected with GIP/ICG@P/R8 NPs of 50 μL (60 nmol kg^{-1} for GIP, 0.5 mg mL^{-1} for ICG) at the inguinal site and imagined at different times of day 1, 2, 3, 4, 5, 6 to detect the fluorescence intensity of each mouse to get the metabolic duration of

GIP/ICG@P/R8 NPs. In order to explore the biodistribution of GIP/ICG@P/R8 NPs, mice were injected with GIP/ICG@P/R8 NPs of 50 μL (60 nmol kg^{-1} for GIP, 0.5 mg mL^{-1} for ICG) at the inguinal site, then ex vivo inguinal WAT, epididymis WAT, interscapular BAT, kidney, heart, lung, liver, spleen were imagined at different time (5 min, 1, 6, 12, 24, 48, 72 h).

1.13 Systematic effects of DIO mice

After the weight of HFD mice was 1.2 times that of normal diet mice, the DIO mice were divided into four groups (n = 8) according to the principle of advanced initial weight. (1) Blank group, the mice were subcutaneously injected in the inguinal region of PBS, (2) ICG@P/R8 NPs group, the mice were subcutaneously injected in the inguinal region of ICG@P/R8 NPs (ICG, 0.5 mg mL^{-1}), and irradiated by a NIR-I laser (808 nm, 0.3 Wcm^{-2}) for 5 minutes, (3) GIP@P/R8 NPs group, the mice were subcutaneously injected in the inguinal region of GIP@P/R8 NPs (GIP, 60 nmol kg^{-1}). (4) GIP/ICG@P/R8 NPs group, the mice were subcutaneously injected in the inguinal region of GIP/ICG@P/R8 NPs (ICG, 0.5 mg mL^{-1}, GIP, 60 nmol kg^{-1}), and irradiated by a NIR-I laser (808 nm, 0.3 Wcm^{-2}) for 5 minutes once a day for 14 days. On the last day, the mice were fasting for 12 hours overnight, weighed, and anesthetized with isoflurane. Then blood was collected from mice heart. Serological parameters (GLU, TG, TC, LDL, and insulin resistance index) were evaluated by a colorimetric quantitative kit (Biosino). The WAT from both sides of the inguinal and epididymis, BAT of the interscapular region were completely removed and weighted. Then the adipose tissues were rinsed with PBS, placed in EP tubes, frozen with liquid nitrogen, and stored in a − 80 °C refrigerator, or fixed with 4% paraformaldehyde solution and stored in a 4 °C refrigerator for further detection.

To investigate the weight loss efficacy of the drug under normal dietary conditions during the treatment period, as well as the individual effects of ICG, NIR, and blank NPs without GIP/ICG, we conducted second and third experiments respectively, following the same experimental protocol as described previously.

1.14 H&E staining and immunofluorescence images

Upon completion of the experiments, major organs (liver, renal, spleen, lung, heart) and different adipose tissue were collected from the DIO mice. These sections were stained with hematoxylin and eosin (H&E) and observed by the microscope photographs.

Adipose tissue was washed by PBS, and fixed in 4% PFA for 15 min at room temperature, after washing, it was permeabilized with PBS + 0.1% TritonX-100 for 5 min at room temperature. Next, it was blocked with 10% serum for 30 min at room temperature after PBS washes. Incubated it with UCP1, PPARγ primary antibody (Proteintech, 16643-1-AP and 23673-1-AP) overnight at 4 °C and goat anti-rabbit secondary antibodies for 1 h in the dark (Abcam, ab150079), adding DAPI for 10 min with washes, then images were collected under microscopy.

1.15 Biocompatibility of GIP/ICG@P/R8 NPs

C57BL/6 male mice purchased from Charles River aged 6 weeks. After 14 days administration of GIP/ICG@P/R8 NPs and NIR exposure for 5 minutes once a day, the blood was collected from the eyeball to examine serum liver function index (AST, ALT), renal function index (CREA, UREA) and blood routine index by a colorimetric quantitative kit (Biosino). The mice subcutaneously injected with PBS were used as the control.

1.16 Statistical Analysis

All experiments were performed at least three times, quantitative results are presented as the mean ± standard deviation (mean ± SD), indicated by error bars in all graphs. Statistical analyses were conducted using GraphPad Prism 9.5 and Origin 2024. Unpaired two-tailed Student’s t-test were used when two groups were compared. One-way analysis of variance (ANOVA) was used to compare the mean values of three or more groups with one independent variable. Two-way ANOVA was used to compare the mean values three or more groups with two independent variables. A P

value of less than 0.05 was considered statistically significant (*P < 0.05, **P < 0.01, ***P < 0.001, ****P<0.0001).

2. Supplementary Figures

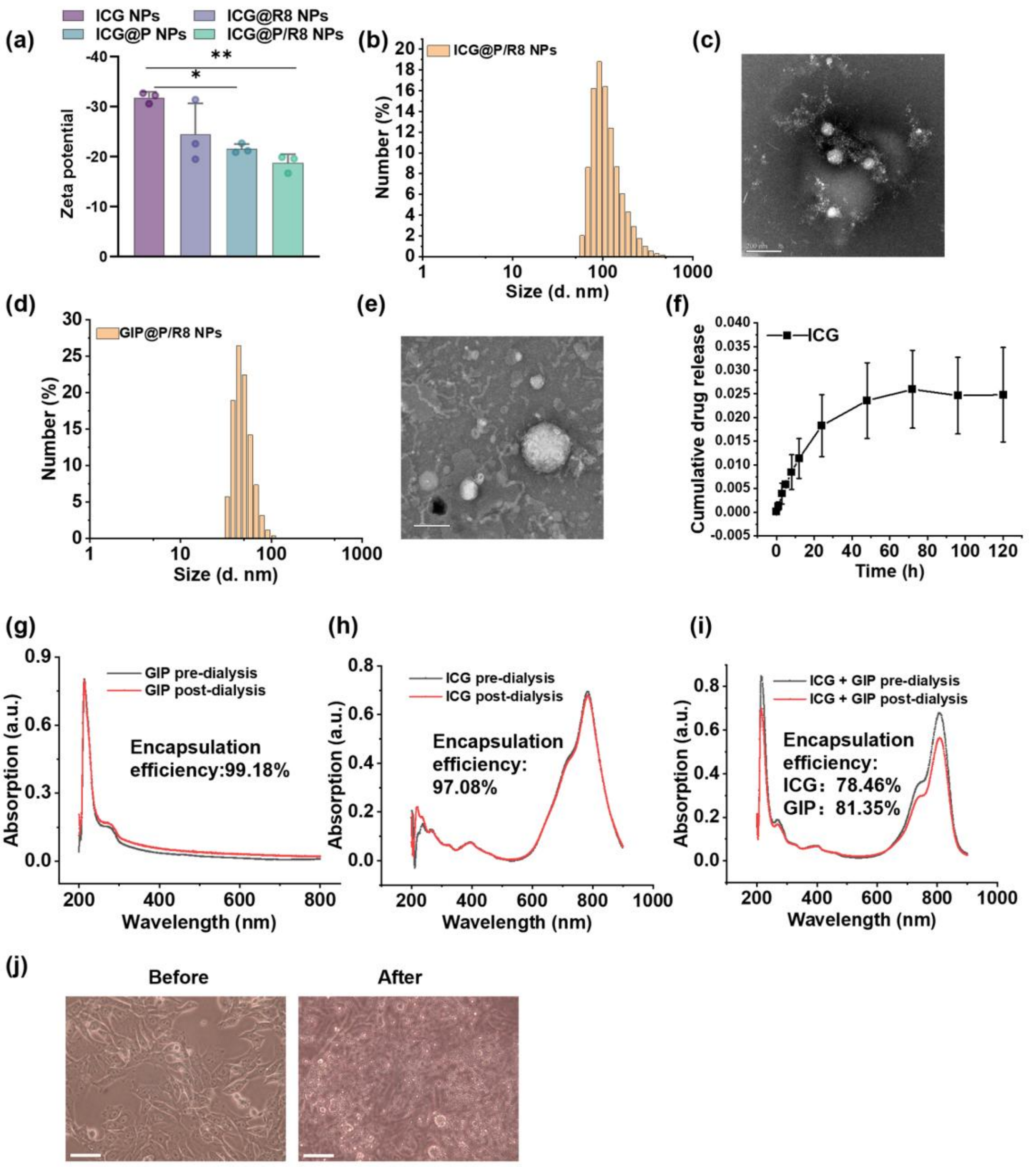

Supplementary Figure 1. (a) The zeta potential of ICG, ICG@R8, ICG@P, and ICG@P/R8 NPs (n = 3). (b, d) The size of (b) ICG@P/R8 and (d) GIP@P/R8 NPs determined by dynamic light scattering. (c, e) Transmission electron microscopy images of (c) ICG@P/R8 and (e) GIP@P/R8 NPs. Scale bar = 200 nm. (f) Time-dependent release profiles of ICG in ICG@P/R8 NPs (n = 3). (g-i) UV-vis-NIR

spectrum of GIP@P/R8, ICG@P/R8 and GIP/ICG@P/R8 NPs before and after dialysis. (j) The different morphology of 3T3L1 preadipocytes before and after differentiation. Scale bar, 50 μm. Data represent the mean ± SD (a, n=3); *$P < 0.05$, **$P < 0.01$ by one-way ANOVA (a).

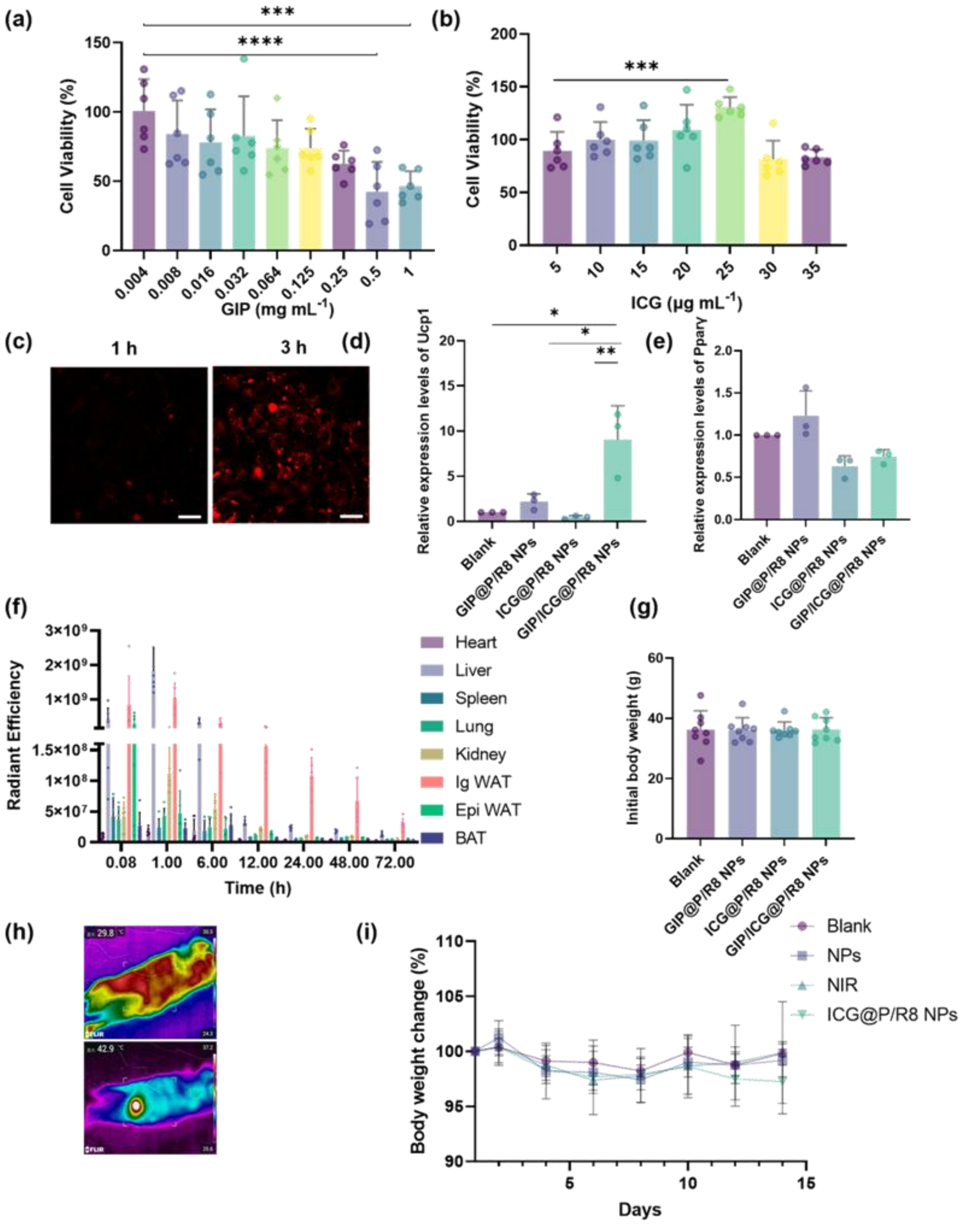

Supplementary Figure 2. (a, b) Cytotoxicity test of GIP@P/R8 NPs and ICG@P/R8 NPs (n = 6). (c) Laser scanning confocal microscopy of mature adipocytes treated with ICG@P/R8 NPs of different time (ICG, red fluorescence). (d, e) Relative gene expression levels of Ucp1 (d) and Pparγ (e) (n = 3). (f) Biodistribution of different organs and time with GIP/ICG@P/R8 NPs (n = 6). (g) Initial weight of mice (n = 8). (h) Infrared thermal images before (top) and after (bottom) the NIR-I laser irradiation. (i) Body weight change after 14 days medication of NIR-only, ICG-only without NIR

and NPs (without GIP/ICG) -only (n = 5). Data represent the mean ± SD; *P < 0.05, **P < 0.01, ***P < 0.001, ****P<0.0001 by one-way ANOVA (a-b, d-e, g), two-way ANOVA (f, i).

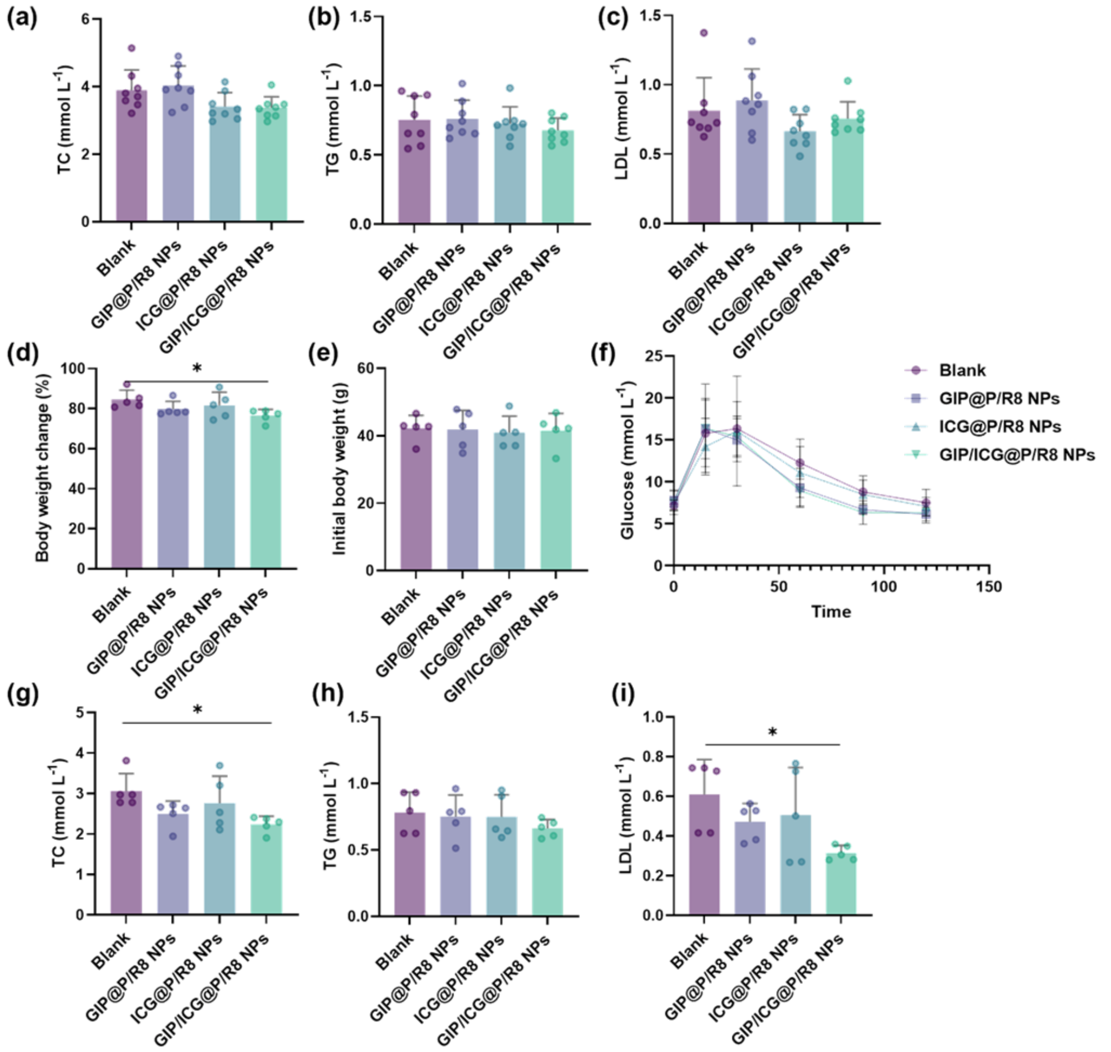

Supplementary Figure 3. (a-c) TC (a), TG (b), and LDL (c) level after 14 days medication continued to HFD (n = 8). (d) Relative body weight change of mice in normal diet after 14 d medication (n = 5). (e) Initial weight of mice in normal diet (n = 5). (f) Oral glucose tolerance test (OGTT) after medication (n = 5). (g-i) TC (g), TG (h), and LDL (i) level after 14 days medication fed on normal diet (n = 5). Data represent the mean ± SD; *P < 0.05, **P < 0.01, ***P < 0.001, ****P<0.0001 by one-way ANOVA (a-e, g-i), two-way ANOVA (f).

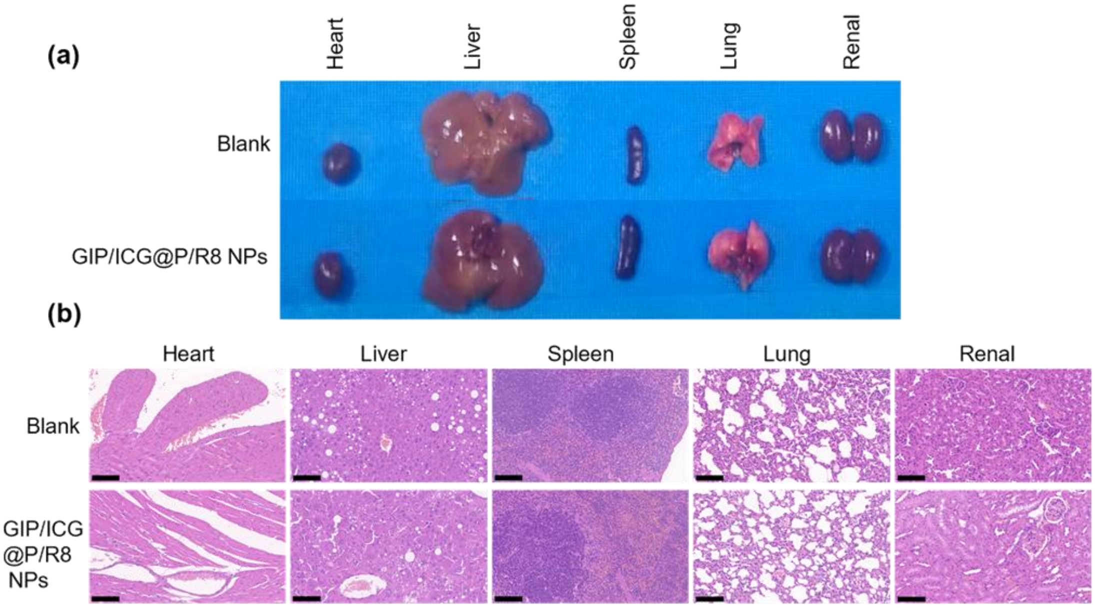

Supplementary Figure 4. (a) The general morphology of different organs of heart, liver, spleen, lung and renal after 14 days medication. (b) H&E staining of heart, liver, spleen, lung and renal. Scale bar, 100 μm.
